# Bee Venom Acupuncture for Neck Pain: A Review of the Korean Literature

**DOI:** 10.3390/toxins15020129

**Published:** 2023-02-04

**Authors:** Soo-Hyun Sung, Hee-Jung Lee, Ji-Eun Han, Angela Dong-Min Sung, Minjung Park, Seungwon Shin, Hye In Jeong, Soobin Jang, Gihyun Lee

**Affiliations:** 1 Department of Policy Development, National Institute of Korean Medicine Development, Seoul 04554, Republic of Korea; 2 Center for Development of Innovative Technologies in Korean Medicine, National Institute of Korean Medicine Development, Seoul 04554, Republic of Korea; 3 Department of Korean Medicine, Graduate School, Kyung Hee University, Seoul 02447, Republic of Korea; 4 Department of Preventive Medicine, College of Korean Medicine, Kyung Hee University, Seoul 02447, Republic of Korea; 5 Department of Preventive Medicine, College of Korean Medicine, Daegu Haany University, Gyeongsan 38609, Republic of Korea; 6 College of Korean Medicine, Dongshin University, Naju 58245, Republic of Korea

**Keywords:** bee venom, bee venom acupuncture, neck pain, clinical studies, traditional Korean medicine

## Abstract

Bee venom is a natural toxin that is effective in treating various types of pain. The purpose of this paper was to review all the features of clinical studies conducted on bee venom acupuncture (BVA) for the treatment of neck pain in Korean publications. Six Korean databases and 16 Korean journals were searched in August 2022 for clinical studies on BVA for neck pain. We identified 24 trials that met our inclusion criteria, of which 316 patients with neck pain were treated with BVA. The most common diagnosis in the patients with neck pain was herniated intervertebral discs (HIVDs) of the cervical spine (C-spine) (29.2%), and the concentration and dosage per session were 0.05–0.5 mg/mL and 0.1–1.5 mL, respectively. The visual analog scale was most often measured for neck pain severity (62.5%), and all clinical research reported improvements in 16 outcome measures. This study shows that BVA could be recommended for the treatment of neck pain, especially HIVD of the C-spine; however, the adverse effects of BVA must be examined in future studies.

## 1. Introduction

Neck pain refers to tension or pain caused by muscle tension or muscle veins in the neck or occipital region, which may limit the range of motion (ROM) in the neck and cause local tenderness and pain radiating to the shoulder blades and upper extremities [1,2]. Compared with other vertebrae, the cervical spine has a relatively large range of motion and structurally weak joint stability owing to its anatomical characteristics [3]. It has been reported that about 67% of the world’s population will experience neck pain at least once in their lifetime, and if it becomes chronic, it can cause serious reductions in quality of life [4]. Neck pain is the most common muscle pain at home and worldwide [4]. Bee venom acupuncture (BVA) involves the injection of purified and diluted bee venom (BV) into acupoints.

The main components of BV are melittin, adolapamin, apamin, and mast cell degranulating peptide [5,6]. It also contains non-peptide components, enzymes, and biologically active amines. The enzymes consist of acid phosphomonesterase, hyaluronidase, lysophospholipase phospholipase A2, and α-d-glucosidase as well as non-peptides such as dopamine, histamine, and norepinephrine [7].

BV is known to have several effects such as immune system activation, anti-inflammation, cytolysis, and radiation protection, and it has been experimentally reported to have anti-inflammatory, analgesic, antipyretic, and anticonvulsant effects. Through this action, it is used in musculoskeletal, purulent, and autoimmune diseases and cancer [8,9]. Practitioners in East-Asian countries (e.g., the Republic of Korea, China, etc.) usually use BV for pharmacopuncture therapy, that is, a combined therapy of herbal medicine and acupuncture [8]. A national survey of traditional Korean medicine (TKM) reported that 22.4% of patients visiting TKM clinics are treated with pharmacopuncture, and BVA is the second most common type of pharmacopuncture in Korea [10,11]. BVA may be accompanied by a rash, itching, chills, fever, vomiting, and diarrhea due to anaphylaxis. In severe cases, it may be accompanied by fainting, breathing difficulty, and even airway obstruction; therefore, this therapeutic method should be administered with care [12,13,14].

Studies on treatment classification for patients with acute neck pain are lacking, and most of them depend on physical therapy at a hospital or conservative treatment [15]. Generally, conservative methods are used to treat chronic neck pain, such as drug treatments, injections at trigger points, massage, and physical therapy. Although a consensus on the treatment has not been reached, demand has continued to increase [1,16,17].

There are many treatment modalities for neck pain, including conventional medicine (CM) treatments (e.g., drugs, physical therapy, etc.). In countries where CM is mainly used, complementary and alternative medicine (CAM) is mostly an additional treatment that supports conventional medicine; however, in East-Asian countries, CAM is often utilized in primary care [18]. Two clinical studies of international databases (the Cochrane Central Register of Controlled Trials, PubMed, and Embase) have examined the clinical effectiveness of BVA for neck pain.

A review of BVA for neck pain has never been published. One randomized controlled trial (RCT) for a neck pain condition was included in a review of musculoskeletal conditions [19]. Korean clinical studies based on TKM treatments have commonly been published in Korean journals rather than in international journals such as the Cochrane Central Register of Controlled Trials, PubMed, and Embase [20]. Therefore, the aim of this study was to investigate TKM clinical trials of BVA for neck pain and to provide clinical evidence for recommending BVA as one of the treatments for neck pain.

## 2. Results

### 2.1. Study Description

**Figure 1:**
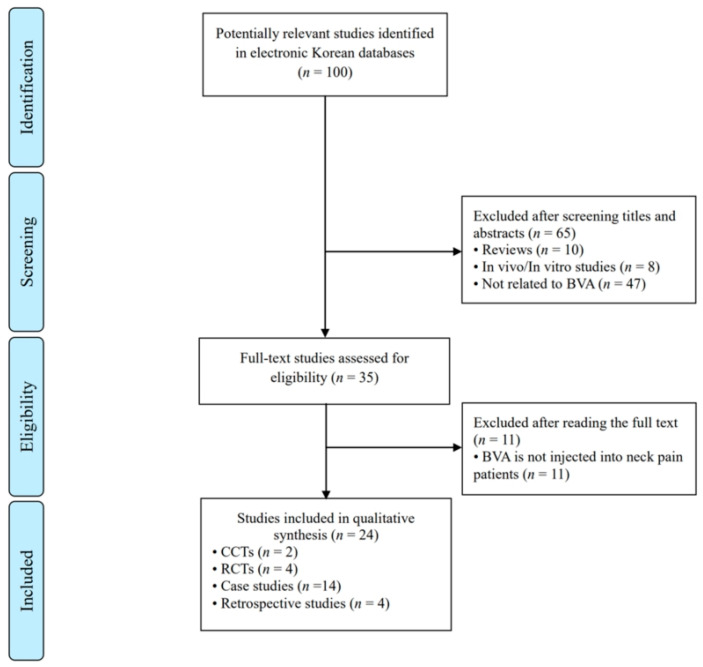
Flowchart of Korean clinical article selection process. BVA: Bee Venom Acupuncture; CCTs: Controlled Clinical Trials; RCTs: Randomized Controlled Trials.

**Figure 2:**
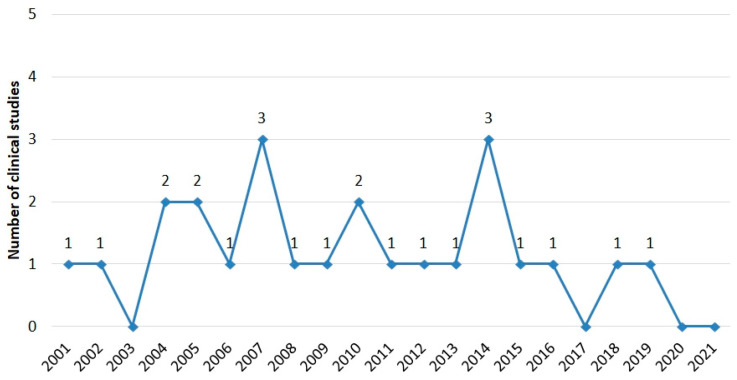
Trend of Korean clinical studies by year.

We identified 25 full-text papers [21,22,23,24,25,26,27,28,29,30,31,32,33,34,35,36,37,38,39,40,41,42,43,44] that met our inclusion criteria (Figure 1). The first clinical trial of BVA for neck pain in Korea was published in 2001. From 2001 to 2020, 0 to a maximum of 3 trials were performed each year, and no clinical trials were conducted in 2003, 2017, 2020, and 2021 (Figure 2). This report includes 14 case studies (58.3%), 2 case-controlled trials (CCTs) (8.3%), 4 RCTs (16.7%), and 4 retrospective studies (16.7%) (Table 1).

### 2.2. Medical Conditions

In total, 12 types of medical conditions were presented in the 25 studies. Seven medical conditions, including herniated intervertebral discs (HIVDs) of the C-spine (28.0%), whiplash injury (12.0%), thoracic outlet syndrome (8.0%), soft tissue damage (8.0%), and neck pain after a car accident (8.0%), were reported in more than two studies. Table 2 shows the numbers of articles and patients according to the medical conditions.

### 2.3. Sample Size

A total of 316 neck pain patients from 24 articles were included in the study.

The sample sizes of the included studies ranged from 1 to 48.

### 2.4. BVA Treatment

BVA was implemented in an injection form in all of the included studies, meaning that practitioners injected BV into acupoints using a syringe. The concentration of the BVA ranged from 0.05 to 0.5 mg/mL for patients with HIVD of the C-spine, and the amount of BV used on the patients varied from 0.1 to 1.0 mL per session and from 0.11 to 27 mL for the entire treatments. The BVA concentrations and dosages for the medical conditions of patients (whiplash injury, soft tissue damage, stiffness of neck, and car accident) are presented in Table 3. Three studies did not mention the concentrations of BVA, six did not mention the dosage for one treatment, and sixteen did not mention the total dosage.

### 2.5. Outcome Measures

**Figure 3:**
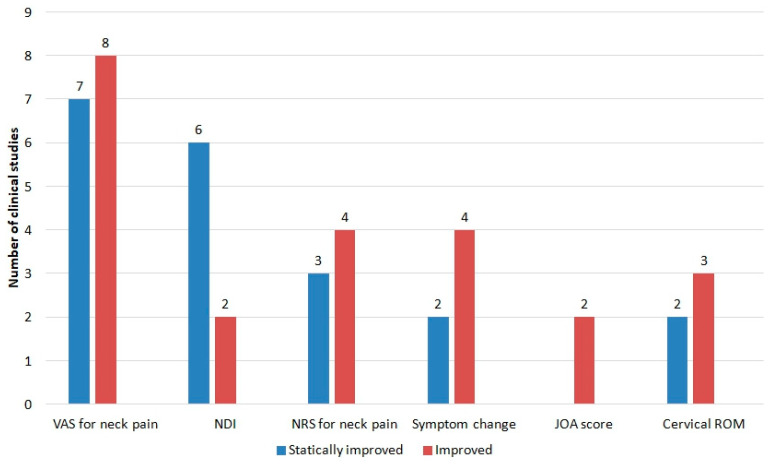
Outcomes of Korean studies of bee venom acupuncture for neck pain. JOA score: Japanese Orthopedic Association assessment treatment score, NRS: numeral rating scale, ROM: range of motion, VAS: visual analog scale.

In total, 16 types of outcomes were measured in the 24 clinical studies (Table 1). The results measured by each outcome were classified into “statistically improved,” “improved,” and “not improved.” The visual analog scale (VAS) was most frequently used to assess neck pain severity (n = 15, 62.5%) (Figure 3). All evaluation tools reported “improved” or “statistically improved” outcome, and none of them reported outcomes that were “not improved.” The case studies and retrospective studies compared before and after treatments to derive statistical significance (statistical improvement). The CCTs and RCTs compared the BVA group and the control group to derive statistical significance (statistical improvement).

## 3. Discussion

This study was conducted to analyze the research pattern and usage of BV in Korean clinical studies. A total of 24 articles based on BVA treatments for neck pain were identified. Among them, four were RCTs and the rest were CCTs (2), case studies (15), and retrospective studies (4). Since 2001, up to three studies have consistently been published every year, all of which reported the positive effects of BVA on neck pain. According to a literature review in 2014, clinical studies have continued to increase since 2000, surpassing experimental studies, while experimental studies have been declining. Notably, RCTs with high levels of evidence have not been reported since 2014, which seems to be the result of the Good Clinical Practice Guidelines (CPG) for Clinical Trials published by the Ministry of Food and Drug Safety (MFDS) [45].

The pain induced by musculoskeletal disorders is generally evaluated subjectively by patients with self-reported outcome measures (e.g., numerical rating scale and VAS). These outcomes of pain severity are regarded as the primary outcomes of neck pain and are appropriate to provide patient satisfaction results to TKM doctors and clinical evidence. The Neck Disability Index (NDI), created in 1991, is a representative questionnaire for evaluating cervical pain that consists of 10 items [46,47]. The Korean version of the NDI was reported in 2009, and the correlation coefficient of its test–retest reliability was 0.927 [48]. There are other questionnaires for evaluating cervical pain: the Neck Pain and Disability Scale, the Cervical Spine Outcome Questionnaire, Patient-Specific Functional Scale self-reports with Neck Dysfunction, and the Copenhagen Neck Functional Disability Scale [48]. Biomarkers for inflammation (e.g., C-reactive protein and interleukin-6 (IL-6)) are also utilized to measure pain. In addition to physical function, psychological function, quality of life, and painkiller dosage can be used as indicators [49].

### 3.1. Current Status of BVA Production in Korea

In Korea, pharmacopunctures containing BVA are prepared at external herbal dispensaries (EHDs) with a good manufacturing practice (GMP) level [50]. We presented a figure of the preparation process of animal venom acupuncture in a previous study [51]. An EHD is a pharmacy that provides TKM clinics with various types of herbal medicines and pharmacopunctures in Korea [52,53]. These EHDs were institutionalized in 2008 and are licensed and managed by the Ministry of Health and Welfare (MoHW) [54]. As a result of a survey in 2019 by the MoHW, BV ampules (vials) were found to be the second most (13.3%) produced pharmacopuncture [55]. As such, the BVA used in Korea is not a drug manufactured by the MFDS. It is prepared in a facility (EHD) that was approved by the MoHW and is used in TKM clinics [56]. In the future, it is expected that research on the use of natural toxin medicines, including the BVA used in Korea, will be activated. In addition, we hope that our results will be used as basic data for the development of natural toxins.

### 3.2. BVA Treatment in TKM Clinics

Pharmacopunctures prepared from EHD are distributed to TKM clinics and hospitals and are utilized for the treatment of various diseases [50]. Based on a national survey of TKM, BVA has mostly been used to treat musculoskeletal disorders (62.3%) [10]. BVA has been reported to be effective for neck pain with all evaluation tools because acupuncture is commonly used to treat musculoskeletal disorders [8,57,58,59,60,61,62,63], including neck pain. BVA plus NASIDs showed significant effects in the outcomes of bothersomeness, pain intensity, and functional status compared with a sham BVA plus NASIDs group [64,65]. Thus, it can be considered that clinical studies on neck pain have been published steadily. In addition, the fact that pharmacopuncture was recognized as a Korean medical practice through the authentic interpretation of the Ministry of Health and Welfare in 1998 may have contributed to the increase in its use [8].

### 3.3. BVA Treatment Standardization Based on TKM CPG

Few studies have examined the optimal dose of BVA for treating neck pain. BVA has become one of the most commonly utilized pharmacopuncture treatments in TKM institutions. BVA is an acupuncture treatment that injects BV into the bodies of patients using a syringe [66,67]. A survey of 393 Korean medicine doctors in 2018 showed that approximately 30% of the respondents used BVA in their clinical practice, which was ranked first among all types of pharmacopuncture [67]. In addition, 38.9% (14 out of 36) of clinical practice guidelines (CPG) of TKM officially accredited and released in Korea include recommendations for BVA, most of which is used for pain alleviation in musculoskeletal disorders [68,69,70,71,72,73,74,75,76,77,78,79,80,81]. However, data on permitted dose information in certifications are limited. Even in the CPG of TKM, only four guidelines recommend BV therapies with specific dose regimens (Table 4). Therefore, we need more evidence of the optimal dose to develop, obtain an authority’s approval, and clinically apply the medical products of BVA. Future follow-up studies such as dose-considered surveys, clinical trials, and a practice-based research network are needed.

### 3.4. Adverse Events of BVA

Only two studies [31,32] reported mild side effects of BVA treatment. In a study by Kim et al. [82], 16.7% of the patients in the BVA group experienced mild to severe symptoms. BV is an animal venom, and side effects may occur when it is applied to the human body. In Korea, when a patient first receives BVA at a TKM clinic, a skin test is mandatory in most cases [67]. According to a pharmacopuncture textbook, patients must be tested for allergies before treatment [8]. This is because the prevention of unexpected immune reactions, such as anaphylaxis, is possible through a skin test prior to treatment [83,84,85,86,87,88]. After subcutaneously injecting 0.1 mL of BV into the skin on the inside of the arm, the injection site was marked and observed for approximately 10 to 15 min [89,90]. The severity and frequency of side effects of BVA for neck pain are currently unclear; therefore, a systematic investigation into the safety of BVA is required.

BV has been used as a drug for a long time, and it is a drug that is widely and legally used in clinics by TKM doctors [51]. There is also a drug approved by the Ministry of Food and Drug Safety with the same ingredients that doctors can use [91]. In particular, melittin, one of the main components of BV, is a controversial ingredient [92]. It is specialized in non-specific cytolytic activity, so recent studies reported its possible adverse effects and how to overcome them [93,94]. The cytotoxic effect can be useful in antitumor methods, but it can interrupt the other purpose, therapeutic application [95]. The natural form of melittin induces non-specific cell lysis and toxicity, so studies on mutation and fusion proteins to decrease the toxicity have been conducted [96].

### 3.5. Study Limitations

This review had several limitations. First, the included studies were mostly case studies with relatively small numbers of the samples. Moreover, it is well-known that case studies are located in a low-quality position in the hierarchy of the evidence pyramid. Therefore, high-quality clinical evidence, such as from randomized controlled trials, is required. Second, the statistical significance of VAS and NDI before and after treatment was reported; however, the measured values were not reported, and a meta-analysis was not conducted. Third, only clinical studies conducted and retrieved in Korea were included in the systematic review; however, those published in international journals (e.g., PubMed, Embase, and the Cochrane Central Register of Controlled Trials) might have been omitted in this review. Fourth, it would be better to report the injection site for the BVA. Nonetheless, this review provides comprehensive information about BV toxins in clinical fields. Additionally, the BVA details provided in this review will help in planning clinical trials for the development of new drugs for neck pain.

### 3.6. Future Suggestion

During BVA treatment, a patient’s typical constitution and condition are diagnosed, and a specific amount of BV is injected into acupoints. BVA provides an immediate effect after treatment because BV is directly absorbed without passing through the gastrointestinal tract [97]. Based on clinical experience, the Korean Pharmacopuncture Institute suggested that BVA can be used for various diseases such as guanwasa, pain, inflammatory joint disease, mental disease, gynecological disease, and brain and cardiovascular disease (Table 5) [8]. In the future, referring to the diseases presented in Table 5, it is expected that evidence-based treatments using BVA will be achieved by verifying its clinical effectiveness and safety based on high-quality multicenter clinical studies.

## 4. Conclusions

This study reports that BVA is being used in TKM clinics and has therapeutic effects in the treatment of neck pain in various diseases. In South Korea, BVA is manufactured in an EHD equipped with GMP-level facilities, and it is diluted and used in the form of injections in the clinical field. In addition, most patients receive treatment after confirming an allergic reaction to bee venom through a skin test before treatment. However, for the potential drug development and clinical application of BVA, identifying its concentration, dosage, treatment sessions, and side effects is necessary.

## 5. Materials and Methods

### 5.1. Data Sources and Searches

We searched KoreaMed (https://koreamed.org), RISS (http://www.riss.or.kr), the National Library of Korea (https://www.nl.go.kr), the Korea Institute of Science and Technology Information (https://scienceon.kisti.re.kr), OASIS (https://oasis.kiom.re.kr), and the Korean Traditional Knowledge Portal (https://www.koreantk.com) from inception to September 2022. All databases were accessed on 15 September 2022. The search terms used for English were as follows: (“bee venom” OR “bee toxin” OR “apitherapy” OR “bee venom therapy” OR “bee venom acupuncture”) AND (“neck pain”) AND (“clinical studies” OR “clinical trial”). In Korean, the search terms were (봉독 OR 봉독침 OR 봉독약침 OR 봉침 OR 봉약침 OR 봉침술) AND (“목 통증” OR 목통증 OR “경추 통증” OR 경추통) AND (임상연구 OR “임상 연구”).

### 5.2. Study Selection

We selected all types of clinical research, including RCTs, retrospective studies such as CCTs, and case studies, that explored the effectiveness of BVA for neck pain. There were no restrictions on the age or gender of patients with neck pain. All types of outcomes (e.g., pain score, Neck Disability Index, ROM, computed tomography, symptom severity change, quality of life, and adverse events) were considered, but they had to be related to the neck pain condition. We did not include in vitro or in vivo experimental studies and reviews.

### 5.3. Data Extraction

Two independent researchers (H.-J.L. and J.-E.H.) sorted the data with a predefined form. The characteristics of the included clinical studies were analyzed, and data were collected on references, study design, sample size, medical conditions, BVA interventions, side effects, outcomes, and major results. When a published paper did not provide sufficient outcome data, we requested raw data via e-mail. Disagreements were settled by discussion with the corresponding author (G.L.).

## Figures and Tables

**Table 1:** Characteristics of Published Clinical Studies in the Korean Literature.

Reference	Study Design/Number of Patients	Medical Conditions	Intervention (Concentration, Treatment Sessions, and Dosage)	Adverse Events	Outcome Measures	Main Result
Kim et al. 2001 [21]	Case study/*n* = 1	HIVD of C-spine in patient with neck pain	0.5 mg/mL	n.r.	VAS for neck pain Cervical ROM C-spine CT (degree of HIVD)	Improved Improved Improved
			1 session: 0.2–1.5 mL			
			Total in 12 sessions: 13.1 mL			
Kang et al. 2002 [22]	CCT/*n* = 17	Soft tissue damage in patients with neck pain	0.1 mg/mL	n.r.	VAS for neck pain Cervical ROM Symptom change	Positive ^a^ Positive ^a^ Improved
			1 session: 0.04–0.09 mL			
			Total in 2 sessions: 0.08–0.18 mL			
Kwon et al. 2004 [23]	CCT/*n* = 10	Soft tissue damage in patients with neck pain	0.3 mg/mL	n.r.	VAS for neck pain Symptom change	Positive ^b^ Positive ^a^
			1 session: 0.05–0.25 mL			
			Total in 2–13 sessions: 0.1–3.25 mL			
Song et al. 2004 [24]	Case study/*n* = 1	Barre–Lieou syndrome in patient with neck pain	0.01 mg/mL or 0.1 mg/mL	n.r.	VAS for neck pain PRS for neck pain	Improved Improved
			1 session: 0.1 mL (0.01 mg/mL) or 0.1–0.3 mL (0.1 mg/mL)			
			Total in 5 sessions: 0.1 mL (0.01 mg/mL) or 1.0 mL (0.1 mg/mL)			
Kim et al. 2005 [25]	RCT/*n* = 10	Sprain of C-spine in patients with neck pain	0.3 mg/mL	n.r.	VAS for neck pain NDI	Positive ^b^ Positive ^b^
			1 session: 0.1 mL			
			Total in 3 sessions: 0.3 mL			
Lee et al. 2005 [26]	Case study/*n* = 14	Cervical radiculopathy in patients with neck pain	0.25 mg/mL or 0.1 mg/mL	hypersensitivity rx, delayed rx, fever	VAS for neck pain JOA score Symptom change	Positive ^c^ Positive ^b^ Positive ^b^
			1 session: 0.1–1.0 mL			
			Total sessions: n.r.			
Kim et al. 2006 [27]	Case study/*n* = 1	Whiplash injury in patient with neck pain	0.03 mg/mL	n.r.	VAS for neck pain Quality of life (SF-36) (1) Physical functioning (2) Social functioning (3) Role limitation (physical) (4) Role limitation (emotional) (5) Mental health (6) Vitality (7) Bodily pain (8) General health	Improved (1) Improved (2) Improved (3) Improved (4) Improved (5) Improved (6) Improved (7) Improved (8) Improved
			1 session: 0.6 mL			
			Total in 8 sessions: 4.8 mL			
Song et al. 2007 [28]	RCT/*n* = 15	Whiplash injuries in patients with neck pain	104 mg/mL	n.r.	VAS for neck pain Cervical ROM	Positive ^a^ Positive ^a^
			1 session: 0.8–1.0 mL			
			Total in 5 sessions: 4.0–5.0 mL			
Lee et al. 2007 [29]	Case study/*n* = 1	HIVD of C-spine in patient with neck pain	0.05 mg/mL or 1 mg/mL	n.r.	VAS for neck pain Symptom change	Improved Improved
			1 session: 0.1–0.2 mL			
			Total in 11 sessions: 0.11–0.22 mL			
An et al. 2007 [30]	Case study/*n* = 1	Cervical spondylosis in patient with neck pain	0.3 mg/mL	n.r.	VAS for neck pain NDI	Improved Improved
			1 session: 0.04–0.2 mL			
			Total sessions and dosage: n.r.			
						


Lee et al. 2008 [31]	RCT/*n* = 21	Stiff neck in patients with neck pain	0.1 mg/mL	localized edema, itching	VAS for neck pain NDI Symptom change	Positive ^c^ Positive ^b^ Positive ^b^
			1 session: 0.2 mL			
			Total in 1 session: 0.2 mL			
Kim et al. 2009 [32]	Case study/*n* = 1	Patient with neck pain after cervical spine surgery	0.25 mg/mL or 0.125 mg/mL	n.r.	VAS for neck pain	Improved
			1 session: n.r.			
			Total sessions and dosage: n.r.			
Oh et al. 2010 [33]	Case study/*n* = 32	Thoracic outlet syndrome in patients with neck pain	n.r.	n.r.	VAS for neck pain	Positive ^b^
			1 session: 0.6 mL			
			Total in 6 sessions: 3.6 mL			
Shin et al. 2010 [34]	Case study/*n* = 1	HIVD of C-spine in patient with neck pain	0.1 mg/mL	n.r.	JOA score Motor grade Cervical ROM	Improved Improved Improved
			1 session: 0.1–1.0 mL			
			Total sessions and dosage: n.r.			
Lee et al. 2011 [35]	Case Study/*n* = 10	Whiplash injuries in patients with neck pain	0.1 mg/mL or 0.05 mg/mL	n.r.	NRS for neck pain NDI	Improved Improved
			1 session: 0.1–2.0 mL			
			Total in 8 sessions: 0.8–16 mL			
Kang et al. 2012 [36]	Retrospective study/*n* = 15	HIVDs of C-spine in patients with neck pain	0.05 mg/mL, 0.1 mg/mL, or 0.5 mg/mL	n.r.	NRS for neck pain Spurling test	Positive ^b^ Improved
			1 session: 0.4–1.0 mL			
			Total in 5 sessions: 2.0–5.0 mL			
Park et al. 2013 [37]	Retrospective study/*n* = 48	HIVDs of C-spine in patients with neck pain	0.1 mg/mL	n.r.	NRS for neck pain NDI	Positive ^c^ Positive ^c^
			1 session: 0.2–1.0 mL			
			Total sessions and dosage: n.r.			
Kim et al. 2014 [38]	Retrospective study/*n* = 28	HIVDs of C-spine in patients with neck pain	0.1 mg/mL	n.r	NRS for neck pain NDI	Positive ^a^ Positive ^a^
			1 session: 1.0 mL			
			Total in 6 sessions: 6.0 mL			
Lee et al. 2014 [39]	Case study/*n* = 1	Thoracic outlet syndrome in patient with neck pain	0.1 mg/mL	none	NRS for neck pain DITI of the upper extremity	Improved Improved
			1 session: 0.5 mL			
			Total in 21 sessions: 10.5 mL			
Lee et al. 2014 [40]	RCT/*n* = 40	Car accident patients with neck pain	0.1 mg/mL	n.r.	VAS for neck pain NDI Pain threshold measured using pressure algometer	Positive ^c^ Positive ^c^ Positive ^b^
			1 session: 0.2–1.0 mL			
			Total in 3 sessions: n.r.			
Jo et al. 2015 [41]	Case study/*n* = 1	Traumatic brachial plexus injury in patient with neck pain	0.03 mg/mL	n.r.	NRS for neck pain Cervical ROM MMT of the upper extremity	Improved Improved Improved
			1 session: n.r.			
			Total in 67 sessions: n.r.			
Song et al. 2016 [42]	Case study/*n* = 1	Cervical spinal cord injury and neurogenic bladder in patient with neck pain	0.1 mg/mL	n.r.	NRS for neck pain ISNCSCI K-MBI	Improved Improved Improved
			1 session: n.r.			
			Total sessions and dosage: n.r.			
Kim et al. 2018 [43]	Retrospective study/*n* = 18	Car accident patients with neck pain	n.r.	n.r.	NDI Symptom change	Positive ^b^ Improved
			1 session: 0.6 mL			
			Total in 7–11 sessions: 4.2–6.6 mL			
Park et al. 2019 [44]	Case study/*n* = 4	HIVDs of C-spine in patients with neck pain	0.125 mg/mL	n.r.	VAS for neck pain	Improved
			1 session: 0.2–1.0 mL			
			Total in 7–29 sessions: 1.4–27.0 mL			

^a^*p* < 0.05; ^b^ *p* < 0.01; ^c^ *p* < 0.001. C-spine: cervical spine, CT: computed tomography, DITI: digital infrared thermography imaging, HIVD: herniated intervertebral disc, ISNCSCI: International Standards for Neurological Classification of Spinal Cord Injury, JOA score: Japanese Orthopedic Association assessment treatment score, K-MBI: Korean Version of Modified Barthel Index, MMT: manual muscle test, NDI: Neck Disability Index, n.r.: not reported, NRS: numeral rating scale, PRS: pain relief scale, ROM: range of motion, SF-36: 36-Item Short FormHealth Survey, VAS: visual analog scale.

**Table 2:** Numbers of studies and patients by medical condition.

Medical Conditions	Number of Studies *n* (%)	Number of Patients Mean (Range)
HIVD of C-spine	7 (29.2)	14 (1–48)
Whiplash injury	3 (12.5)	16.7 (10–25)
Thoracic outlet syndrome	2 (8.3)	16.5 (1–32)
Soft tissue damaged	2 (8.3)	13.5 (10–17)
Car accident	2 (8.3)	29 (18–40)

C-spine: cervical spine, HIVD: herniated intervertebral disc.

**Table 3:** BVA concentrations and dosages for the medical conditions of patients.

Medical Conditions of Participants	Concentration (mg/mL)	Dosage	
		Dosage per 1 Session (mL)	Dosage for Entire Treatment (mL)
HIVDs of C-spine in patients with neck pain	0.05–0.5	0.1–1.5	0.11–27.0
Whiplash injuries in patients with neck pain	0.03–104	0.4–1.0	0.11–5.0
Thoracic outlet syndrome in patients with neck pain	0.1	0.5–0.6	3.6–10.5
Soft tissue damage in patients with neck pain	0.1–0.3	0.04–0.25	0.08–3.25
Car accident patients with neck pain	0.1	0.2–1.0	4.2–6.6

C-spine: cervical spine, HIVD: herniated intervertebral disc.

**Table 4:** Clinical practice guidelines of traditional Korean medicine in Korea recommending bee venom acupuncture.

Recommendation	Clinical Practice Guidelines
With a specific dose	Ankle sprain [68], unspecific chronic low back pain [69], and knee osteoarthritis [70]
Without a specific dose	Stroke [71], shoulder pain [72], traffic injuries [73], temporomandibular joint disorder [74], neck pain [75], herniation of lumbar disk [76], gout [77], facial palsy [78], tension-type headache [79], cancer-related symptoms [80], and breast cancer [81]

**Table 5:** BVA treatments of diseases recommended by the Korean Pharmacopuncture Institute.

Classification	Specific Disease
Pain	Back pain, knee pain, neck pain, shoulder pain, sciatica, intercostal neuralgia, trigeminal neuralgia, cervical disc herniation, and cervical sprain (whiplash injury)
Inflammation and joint diseases	Temporomandibular joint syndrome, degenerative knee arthritis, knee joint collateral ligament injury, degenerative hip arthritis, knee joint bursitis, ankle sprain, plantar fasciitis, cervical disc herniation, back sprain, spinal stenosis, lumbar disc herniation, compression fracture, frozen shoulder, rotator cuff tear, impingement syndrome, thoracic outlet syndrome, humeral trauma, medial epicondylitis, traumatic synovitis of the elbow, carpal tunnel syndrome, Degure’s disease, radial nerve palsy, trigger finger, ganglionoma, and osteoarthritis of the hand
Cardiovascular diseases	Headache, stroke, vertigo, hypertension, and angina
Digestive diseases	Indigestion, abdominal pain, gastritis, gastric and duodenal ulcers, irritable bowel syndrome, constipation, and diarrhea
Respiratory diseases	Cold, cough after cold, asthma, bronchiectasis, and pulmonary fibrosis
Genitourinary diseases	Prostatitis, vulva, cystitis, urethritis, cystitis, and edema
Systemic and immune diseases	Rheumatoid arthritis, systemic lupus erythematosus, ankylosing spondylitis, polymyositis and dermatomyositis, systemic sclerosis, Sjogren’s syndrome, Behçet’s disease, fibromyalgia, multiple sclerosis, and myasthenia gravis
Endocrine and metabolic diseases	Diabetes, thyroid disease, and obesity
Neurological and psychiatric diseases	Insomnia, depression, and palpitation
Ear, nose, and throat diseases	Dry eye syndrome, acute/chronic rhinitis, allergic rhinitis, atrophic rhinitis, sinusitis, tinnitus, otitis media, nucleolus, myopia, strabismus, and facial nerve palsy
Dermatological diseases	Shingles, psoriasis, atopy, eczema, pompholyx, acne, molluscum contagiosum, athlete’s foot, warts, and alopecia
Gynecological diseases	Dysmenorrhea, vaginal dryness, and menopausal syndrome

## Data Availability

The datasets used and/or analyzed during the current study are available from the corresponding author upon reasonable request.
